# Functional comparison of plasma-membrane Na^+^/H^+ ^antiporters from two pathogenic *Candida *species

**DOI:** 10.1186/1471-2180-8-80

**Published:** 2008-05-20

**Authors:** Yannick Krauke, Hana Sychrova

**Affiliations:** 1 Department of Membrane Transport, Institute of Physiology AS CR, v.v.i., Videnska 1083, 14220 Prague 4, Czech Republic

## Abstract

**Background:**

The virulence of *Candida *species depends on many environmental conditions. Extracellular pH and concentration of alkali metal cations belong among important factors. Nevertheless, the contribution of transporters mediating the exchange of alkali metal cations for protons across the plasma membrane to the cell salt tolerance and other physiological properties of various *Candida *species has not been studied so far.

**Results:**

The tolerance/sensitivity of four pathogenic *Candida *species to alkali metal cations was tested and the role of one of the cation transporters in that tolerance (presumed to be the plasma-membrane Na^+^/H^+ ^antiporter) was studied. The genes encoding these antiporters in the most and least salt sensitive species, *C. dubliniensis *and *C. parapsilosis *respectively, were identified, cloned and functionally expressed in the plasma membranes of *Saccharomyces cerevisiae *cells lacking their own cation exporters. Both *Cp*Cnh1 and *Cd*Cnh1 antiporters had broad substrate specificity and transported Na^+^, K^+^, Li^+^, and Rb^+^. Their activity in *S. cerevisiae *cells differed; *Cp*Cnh1p provided cells with a much higher salt tolerance than the *Cd*Cnh1 antiporter. The observed difference in activity was confirmed by direct measurements of sodium and potassium efflux mediated by these antiporters.

**Conclusion:**

We have cloned two genes encoding putative Na^+^/H^+ ^antiporters in *C. parapsilosis *and *C. dubliniensis*, and characterized the transport properties of encoded proteins. Our results show that the activity of plasma-membrane Na^+^/H^+ ^antiporters is one of the factors determining the tolerance of pathogenic *Candida *species to high external concentrations of alkali metal cations.

## Background

The family of *Candida *species, normally a harmless human commensal of the gastrointestinal and genitourinary tract, can become a human pathogen under certain circumstances. Mainly in HIV and immunocompromised patients, *Candida *cause a wide range of infections and are the most prevalent pathogenic yeast. One key feature of these fungi is their ability to grow in three different morphologies: yeast, pseudohyphae and true hyphae [1]. This reversible switching from one form to another is dependent on environmental conditions like temperature, pH, nutritional status and external/internal concentration of cations [2].

In general, pathogenic *Candida *species are osmotolerant yeasts and can grow, with the exception of *Candida dubliniensis *[3], at relatively high NaCl concentrations, although the presence of salt was shown to negatively influence several virulence traits of *Candida albicans *[4]. Recent experiments also suggest a relationship between the formation of *C. albicans *hyphae and the intracellular concentration of potassium [5]. Nevertheless, the regulation of intracellular potassium and sodium concentrations in *Candida *species has not been studied in detail.

Yeast species in general have several transport systems in their plasma membranes at their disposal to maintain homeostasis in alkali metal cations, i.e. a high ratio between potassium (which is the main intracellular cation) and toxic sodium concentrations [6-8]. Among these transport systems, Na^+^/H^+ ^antiporters play an important role. Most yeasts that have so far been studied (e.g. *Saccharomyces cerevisiae, Debaryomyces hansenii) *possess only one type of this antiporter in their plasma membranes, which efficiently transports both sodium and potassium cations from the cells, as well as their analogues lithium and rubidium [7,9]. A few yeast species (*Yarrowia lipolytica, Schizosaccharomyces pombe) *have two antiporters of this family at their disposal, one of them with a substrate preference for sodium and lithium, the other preferring potassium and rubidium [10,11].

The Na^+^/H^+ ^antiporter that has been studied the most so far is from *S. cerevisiae*, encoded by the *NHA1 *gene, and has 12 predicted trans-membrane domains and a very long hydrophilic C-terminus [7]. Beside its function in removing toxic Na^+ ^from cells and maintaining potassium homeostasis, it is involved in several other cellular functions such as regulating intracellular pH [12,13], cell volume [7], plasma membrane potential [14] and the cell cycle [15,16], and it participates in the cell response to osmotic shock [7,17]. The Nha1p orthologs from *C. albicans *and *Candida tropicalis Ca*Cnh1p and *Ct*Cnh1p, respectively, were functionally characterized upon heterologous expression in *S. cerevisiae*. Both showed the same broad substrate specificity as *Sc*Nha1p [18,19]. The deletion of *CNH1 *in *C. albicans *results in cell sensitivity to high external potassium concentrations [20] and under some conditions causes slight changes in cell morphology [21].

In this work we compared the tolerance of four different pathogenic *Candida *species to alkali metal cations, performed a search for Nha1/Cnh1 antiporter-encoding orthologs in their genomes, and characterized the transport properties of the Na^+^/H^+ ^antiporters from the most and least tolerant species, *C. parapsilosis *and *C. dubliniensis *respectively.

## Results

### *Candida *species differ in their halotolerance

**Figure 1:**
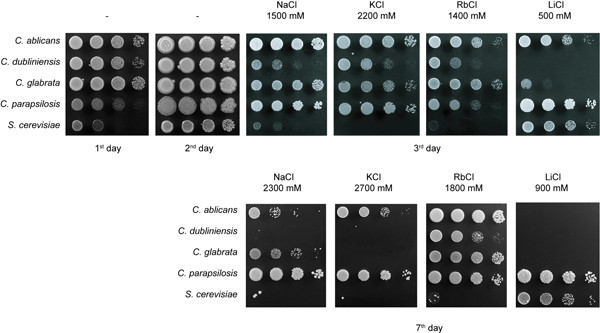
Growth of various *Candida *species and *S. cerevisiae *on YPD medium supplemented with salts at 30°C.

According to the literature [3], *C. dubliniensis *is relatively sodium sensitive, whereas *C. parapsilosis *was shown to tolerate high NaCl concentrations [22]. In order to estimate the tolerance of *Candida *species to different alkali metal cations, the growth of four *Candida *species and a *S. cerevisiae *wild type (as a non-osmotolerant control) in the presence of increasing concentrations of various salts was estimated. In the absence of salts, *S. cerevisiae *cells grew more slowly than all four *Candida *species, and also the growth of *C. parapsilosis *was not as robust as with the other three *Candida *species (Figure 1). All yeast species grew equally well in the presence of lower salt concentrations (cf. Methods) but as the amount of alkali-metal-cation on the plates increased, important differences were observed. Of the tested *Candida *species, *C. dubliniensis *had the lowest tolerance to all of the tested salts, it is not able to grow when the salt concentrations are above 1600 mM NaCl, 2300 mM KCl or 200 mM LiCl. Nevertheless, *C. dubliniensis *is more sodium, potassium and rubidium tolerant than *S. cerevisiae*, but is much more sensitive to toxic lithium cations (Figure 1). Also, the lithium sensitivity of *C. glabrata *is higher than that of *S. cerevisiae *but *C. glabrata *cells can grow in much higher concentrations of the other salts than *S. cerevisiae *cells (up to 2300 mM NaCl, 2400 mM KCl, 1800 mM RbCl; Figure 1). *C. albicans *and *C. parapsilosis *are the most halotolerant species, and *C. parapsilosis *seems to grow even better in the presence of high salt concentrations than *C. albicans *(Figure 1). To summarize, the four *Candida *species have not only different sensitivity to NaCl, as published previously [3,22] but they differ in their tolerance to alkali metal cations in general. *C. albicans *and *C. parapsilosis *are highly halotolerant and *C. dubliniensis *is halosensitive.

### Comparison of *Candida CNH1 *genes and encoded antiporters

A search in databases revealed the existence of open reading frames homologous to the *CaCNH1 *gene in the genomes of *C. dubliniensis, C. glabrata *and *C. parapsilosis*. In these species, just one homologous sequence was found, suggesting that their plasma-membrane Na^+^/H^+ ^antiporters have a broad substrate specificity to alkali metal cations, similar to those of *C. albicans *and *C. tropicalis*. We named the identified orthologous genes according to their species of origin, *CdCNH1, CgCNH1 *and *CpCNH1*. The *CdCNH1 *gene is 2454 nt (818 aa) long, *CgCNH1 *has 2835 nt (945 aa), and the *CpCNH1 *gene is composed of 2955 nt (985 aa). Neither of them have any introns.

The predicted protein structures of these three antiporters were compared with the two *Candida *antiporters that have already been characterized, *Ca*Cnh1 and *Ct*Cnh1. Comparison of the protein length and predicted structure of the Cnh1 proteins from five *Candida *species and *S. cerevisiae *revealed that *Cp*Cnh1p is the longest and *C. dublinienis *antiporter the shortest member of the *Candida *Na^+^/H^+^antiporters' subfamily (Table 1). For all *Candida *proteins, the Kyte-Doolittle method predicted a similar structure to *Sc*Nha1p with highly conserved N-termini and 12 trans-membrane sections (Tables 1 &2). On the other hand, they differ in the length and composition of their hydrophilic C-termini, as do the antiporters from non-*Candida *yeast species [23]. The most significant is the difference in length (approx. 180 aa) between the C-termini of *Cp*Cnh1p (555 aa), *Ca*Cnh1p (366 aa) and *Cd*Cnh1p (388 aa). Of the analyzed proteins, *Ca*Cnh1p and *Cd*Cnh1p show the highest sequence identity in all parts of the protein, though the hydrophobic trans-membrane domains and connecting loops are highly conserved (approx. 90% identity) in all *Candida *antiporters except for *Cg*Cnh1p (Table 2). *C. glabrata *Cnh1p is more similar to *S. cerevisiae *Nha1p than to the antiporters from other *Candida *species in all the features analyzed, which corresponds to the phylogenetic relationships among these yeast species [24].

**Table 1:** Comparison of deduced secondary structures of plasma-membrane Na^+^/H^+ ^antiporters from *Candida *species and *S. cerevisiae*

Yeast	Antiporter	Number of amino acid residues			
	
		N-terminus	Tms + loops	C-terminus	Whole protein
*C. albicans*	*Ca*Cnh1p	11	419	366	796
*C. dubliniensis*	*Cd*Cnh1p	11	419	388	818
*C. glabrata*	*Cg*Cnh1p	12	418	515	945
*C. parapsilosis*	*Cp*Cnh1p	11	419	555	985
*C. tropicalis*	*Ct*Nha1p	11	419	545	975
*S. cerevisiae*	*Sc*Nha1p	12	418	555	985

**Table 2:** Identity (%) of *Candida *and *S. cerevisiae *alkali-metal-cation antiporters

	**Entire protein**/tms and loops/C-terminus					
Antiporter	*Ca*Cnh1p	*Cd*Cnh1p	*Cg*Cnh1p	*Cp*Cnh1p	*Ct*Nha1p	*Sc*Nha1p

*Ca*Cnh1p	**-**	**84.2/**98.6/67.5	**46.0/**69.9/20.5	**64.5/**88.3/39.6	**69.0/**93.1/41.0	**44.0/**68.7/23.5
*Cd*Cnh1p			**46.2/**69.4/22.2	**62.7**/88.1/35.3	**67.5/**93.1/43.8	**46.7/**69.5/19.8
*Cg*Cnh1p				**42.7/**70.1/21.4	**39.7/**69.4/19.6	**57.0/**86.8/31.8
*Cp*Cnh1p					**57.1/**86.8/34.2	**38.8/**68.7/20.6
*Ct*Nha1p						**41.0/**69.4/19.0

Though the highest divergence from identity was found in the C-termini of all the compared antiporters, the existence of six conserved C-terminal regions described previously [18] was also confirmed in *C.dubliniensis, C. parapsilosis *and *C. glabrata *species (not shown). A new, approximately 25 aa-long conserved region (K/R)(L/I)**SR**(S/T)(L/A)**SRRS**(Y/F)**Y**(K/R)**KDDP**(H/N)(K/R)**RKVYAHR **(in *Ca*Cnh1p aa 639–664) preceding conserved region no. 5 [18]; was found in the four *Candida *species, except for *C. glabrata*.

Both the *C. dubliniensis *and *C. parapsilosis *species belong to the group of yeasts in which the CTG codon encodes a serine and not a leucine, as in other yeast species (i.e. *S. cerevisiae*) [25]. One CTG codon exists at aa position 621 in *CpCNH1*. This serine 621 is localized in the antiporter's hydrophilic C-terminus and not in the membrane part of the protein. It is localized in a small weakly conserved area where at a similar position *Cd*Cnh1p (aa 568) and *Ct*Cnh1p (aa 644) also have a serine and *Ca*Cnh1p (aa 552) has an isoleucine.

### Heterologous expression of *Candida *Cnh1ps in *S. cerevisiae *BW31a cells and its phenotype

In order to 1) verify whether the identified and analysed open reading frames encode functional plasma-membrane alkali-metal-cation/proton antiporters, and 2) elucidate whether the species' salt tolerance could reflect the activity of these antiporters, corresponding DNA fragments from the most and least salt tolerant species, *C. parapsilosis *and *C. dubliniensis *respectively, were expressed in a *S. cerevisiae *mutant lacking its own export systems for alkali metal cations (BW31a *ena1-4*Δ *nha1*Δ [8]). The lack of both Na^+^-ATPases, Ena1-4 and the Na^+^/H^+ ^antiporter Nha1 renders these cells extremely sensitive to higher external concentrations of salts, and almost no efflux of sodium or potassium cations from them is observable. The functional expression of heterologous sodium and/or potassium exporters in BW31a cells has clear phenotypes of an increased salt tolerance and measurable alkali metal cation efflux [26].

**Figure 2:**
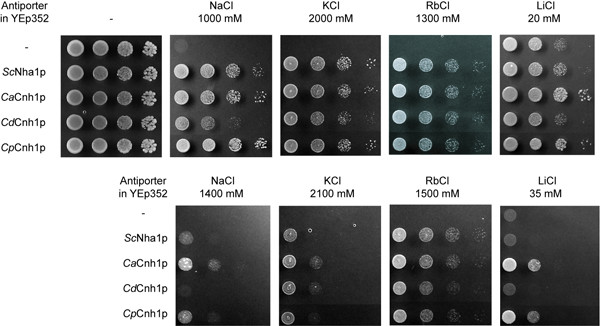
**Growth of *S. cerevisiae *BW31a cells expressing various antiporters**. Cells expressing various antiporters were grown on YNB media supplemented with salts at 30°C. Cells with empty YEp352 vector (-) were used as negative, cells expressing *Sc*Nha1p as positive control, respectively. Pictures were taken after 4 (YNB) or 7 days (YNB + salts) of incubation.

The *CdCNH1 *and *CpCNH1 *genes amplified from genomic DNAs were cloned behind the *ScNHA1 *promoter and expressed from multicopy vectors, as were the genes *ScNHA1 *and *CaCNH1 *[7,19], which served as positive controls in our study. Thus all four antiporters were expressed under the same conditions (strain, vector, promoter) which should ensure similar antiporters' levels in cells. Empty YEp352 and pGRU1 vectors served as negative controls. The functionality of all the constructs were first tested in drop experiments, which showed that 1) the presence of the constructs did not influence the growth rate of cells in standard media, i.e. the heterologous expression of these membrane proteins was not toxic for *S. cerevisiae*, and 2) the expression of both GFP-tagged and non-tagged *Cd*Cnh1 and *Cp*Cnh1 proteins brought about the same ability to grow on 800 mM NaCl or 1800 mM KCl, as did the positive controls with *Sc*Nha1 and *Ca*Cnh1 proteins, whereas the cells without antiporters were not able to grow (not shown). This result also confirmed that the C-terminal GFP-tagging did not influence the activity of the antiporters. In order to estimate the substrate specificity and transport capacity of the antiporters, BW31a cells expressing the four antiporters or transformed with an empty vector were spotted on a series of YNB plates containing increasing NaCl, KCl, LiCl and RbCl concentrations. Cells expressing *Cp*Cnhp1p were able to grow in the highest concentrations of salts, as did cells expressing *Ca*Cnh1p. Both these *Candida *antiporters conferred a slightly higher tolerance to the cells than equivalent expression of the native *S. cerevisiae *antiporter, Nha1p (Figure 2). The tolerance of cells expressing *Cd*Cnh1p to high external potassium and rubidium was almost the same as for cells expressing *Cp*Cnhp1p and *Ca*Cnh1p (Figure 2), but their tolerance to toxic cations was significantly lower, only 1000 mM NaCl, and there was no increase in LiCl tolerance compared to cells with the empty vector (30 mM LiCl in both cases).

The proton-antiport mechanism of these Cnh1 proteins was verified in a series of drop tests on plates with various pH values (Table 3). As was previously thought, the cells expressing antiporters showed the highest salt tolerance (at least for three of their four substrates) when grown at lower external pH, i.e. in conditions where the proton gradient across the plasma membrane is the highest. Surprisingly, the expression of both the *S. cerevisiae *and *C. dubliniensis *antiporters did not increase the cell tolerance to lithium cations at pH 3.5, suggesting that Li^+ ^was not recognized as their substrate under these conditions. On the other hand, all four antiporters were partially active, even at an externally neutral pH 7.0, as their presence enabled the cells to support higher salt concentrations. This detailed study confirmed again that *Cd*Cnh1p has the least ability to improve the salt tolerance of cells (Table 3).

**Table 3:** pH dependence of salt tolerance of BW31a cells expressing various antiporters.

	NaCl (mM)			KCl (mM)			LiCl (mM)			
				
Antiporter	3.5	5.5	7.0	3.5	5.5	7.0	3.5	5.5	7.0	pH_out_
-	<500	200	75	1000	1000	600	35	15	<5	
*Sc*Nha1p	1300	800	150	2000	2000	1200	35	25	7	
*Ca*Cnh1p	1300	1000	200	2000	2000	1200	50	40	10	
*Cd*Cnh1p	1100	800	200	2000	2000	1200	35	25	7	
*Cp*Cnh1p	1300	1000	200	2000	2000	1300	40	30	7	

Maximum salt concentrations [mM] allowing growth of *S. cerevisiae *BW31a cells (transformed with the YEp352 plasmid either empty or harbouring genes encoding antiporters) on YNB media with various pH values.

### Localization of *Cd*Cnh1 and *Cp*Cnh1 antiporters in *S. cerevisiae *cells

**Figure 3:**
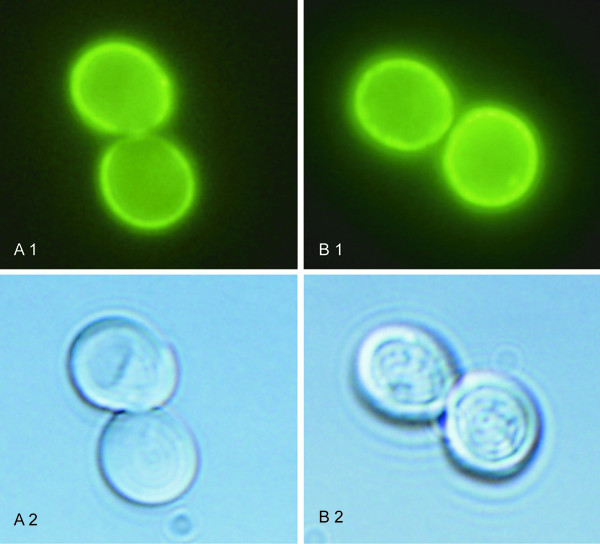
Localization of GFP-tagged *Cd*Cnh1p (A) and *Cp*Cnh1p (B) in BW31a cells. Fluorescence (1) and Nomarski (2) micrographs.

As mentioned above, C-terminal GFP-tagging of the *Cd*Cnh1 and *Cp*Cnh1 proteins did not affected their functionality. Both antiporters improved the cell salt tolerance to a similar degree as the non-tagged versions, and fluorescence microscopy localized them to the plasma membrane of *S. cerevisiae *BW31a cells (Figure 3) and not to the membranes of intracellular organelles. This result indicates a high probability of the same localization in their organisms of origin, as was previously shown for *C. albicans *Cnh1p [20].

### Western blot analysis of antiporters' amount in cells

**Figure 4:**
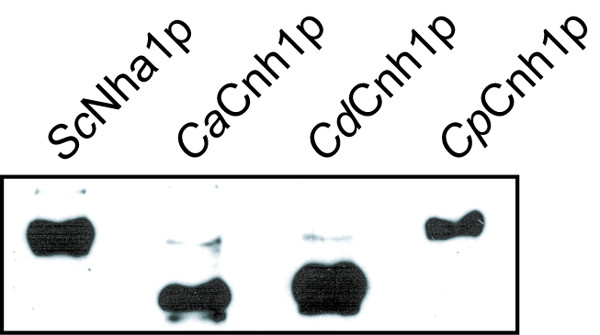
**Immunodetection of GFP-tagged antiporters in *S. cerevisiae *cells**. Cells expressing *Sc*Nha1-GFP, *Ca*Cnh1-GFP, *Cd*Cnh1-GFP and *Cp*Cnh1-GFP were grown to exponential phase and proteins extracted. After separation of proteins via SDS-PAGE they were transferred to nitrocellulose membrane and detected with anti-GFP antibody.

To verify whether the use of the same vector and promoter for expression ensures similar levels of the four antiporters in BW31a cells, the GFP-tagged proteins were visualized on western blots. Fig. 4 shows that 1) the size of *Sc *and *Cp *antiporters was alike, similarly as the size of *Ca *and *Cd *transporters (and in agreement with the size deduced from the gene sequence, cf. Table 1 and 2) the quantity of antiporters in extracts of exponentially growing cells was similar, the highest amount apparently being observed for *Cd*Cnh1p and the lowest one for *Cp*Cnh1 antiporter. The analysis was repeated three times with the same result. The fluorescence microscopy and western blot analysis confirmed that the observed low activity of *Cd*Cnh1p and the high activity of *Cp*Cnh1 antiporter did not reflect different protein levels in the cells.

### Efflux of Na^+ ^and K^+ ^from BW31a cells expressing various antiporters

**Figure 5:**
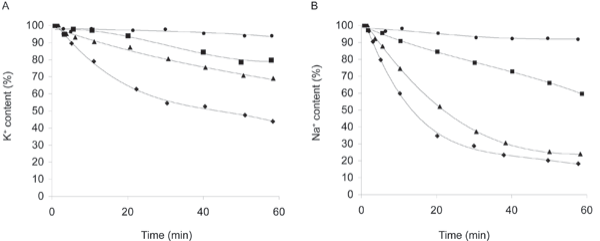
**Efflux of cations from BW31a cells expressing various antiporters**. The efflux of K^+ ^(**A**) and Na^+ ^(**B**) from BW31a cells harbouring the empty plasmid YEp352 (●) or expressing *Cd*Cnh1p (■), *Cp*Cnh1p (▲), *Ca*Cnh1p (♦) antiporters was measured. The initial concentrations of cations in cells (corresponding to 100%) were as follows: Na^+^, 110.5 ± 6.8 nmol (mg dry wt)^-1^, K^+^, 549.5 ± 25.7 nmol (mg dry wt)^-1 ^(representative results are shown).

To confirm the results from drop test experiments and to determine the activity and efflux rate of *Cd*Cnh1p and *Cp*Cnh1p, the efflux of K^+ ^and Na^+ ^was directly measured. The loss of K^+ ^from BW31a cells expressing *C. dubliniensis *or *C. parapsilosis *antiporters was measured directly; to measure Na^+ ^efflux, cell preloading with 100 mM NaCl was necessary (cf. Methods). Cells expressing the *Ca*Cnh1 antiporter served as positive, and cells transformed with an empty vector as negative controls, respectively. The initial internal concentration of K^+ ^in exponentially growing cells was almost the same in all strains, in the representative experiment about 549.5 ± 25.7 nmol (mg dry wt)^-1^. After preloading, cells contained 110.5 ± 6.8 nmol (mg dry wt)^-1 ^Na^+^. As shown in Figure 5 and Table 4, *Cp*Cnh1p exported Na^+ ^and K^+ ^much more efficiently than *Cd*Cnh1p. Within 60 minutes, cells with *Cp*Cnh1p lost 80% of their internal sodium and 32% of their potassium compared to cells with *Cd*Cnh1p, which over the same period only lost 38% of their sodium and 20% of their potassium. The *C. albicans *antiporter was the most effective, exporting 82% of its host cells' sodium and 55% of their potassium in 60 min, which agrees with previously published results [19]. The Na^+ ^efflux curves for *Ca*Cnh1p and *Cp*Cnh1p are very similar and almost exponential; most of the sodium is exported in about 40 minutes. The efflux of sodium via *Cd*Cnh1p is linear and slow. The sodium efflux via all three antiporters is faster than their potassium efflux, though the initial intracellular concentration of K^+ ^is much higher than that of Na^+^, approx. 300 vs. 50 mM. These results suggest that the *Candida *antiporters have, at least upon heterologous expression in *S. cerevisiae*, a higher affinity for sodium than for potassium cations.

**Table 4:** Efflux of potassium and sodium cations from BW31a cells expressing various antiporters.

Antiporter	Na^+^	K^+^
-	7.4 ± 0.7	5.0 ± 1.0
*Ca*Cnh1p	82.2 ± 0.4	55.3 ± 1.8
*Cd*Cnh1p	38.4 ± 3.0	19.5 ± 2.1
*Cp*Cnh1p	79.1 ± 2.8	32.6 ± 3.0

The total loss (%) of Na^+^and K^+^was measured over 60 min. The values are the average of three independent experiments, each consisting of three samples per time point, the initial concentrations of cations were: K^+^, 539.5 ± 29.7 nmol (mg dry wt)^-1^; Na^+^, 118.9 ± 19.1 nmol (mg dry wt)^-1^.

## Discussion and Conclusion

The variable sensitivity of *Candida *species to NaCl has been observed and exploited previously, mainly in connection with the original niche of the species, e.g. the high NaCl tolerance of *C. parapsilosis *strains isolated from sea water or hypersaline brines [22,27] or in clinical microbiological tests aiming to distinguish among *Candida *species (NaCl sensitivity of *C. dubliniensis*[3]). In this work, we performed a series of tests to characterize the tolerance of four different pathogenic *Candida *species to different alkali metal cations, and addressed the question of whether the observed differences in salt tolerance could be based on the transport activity and/or specificity of *Candidas' *plasma-membrane Na^+^/H^+ ^antiporters. Our results showed clearly that, besides differences in their tolerance to sodium, the four species tested also differ in their sensitivity to highly toxic lithium and their tolerance of high external concentrations of non-toxic potassium cations (Figure 1). *C. glabrata *and *C. dubliniensis *are the most sensitive to Li^+ ^cations, the latter being the least alkali-metal-cation tolerant of the species tested.

To elucidate the role of Na^+^/H^+ ^antiporters in *Candidas' *salt tolerance, we isolated and heterologously expressed genes encoding putative antiporters in the most and the least tolerant species, *C. parapsilosis *and *C. dubliniensis*. The functional characterization of these encoded proteins in an *S. cerevisiae *mutant lacking its own alkali-metal-cation exporters revealed that both antiporters have broad substrate specificity. They recognize at least four different cations (K^+^, Li^+^, Na^+^, Rb^+^), but transport them with varying capacities and/or affinities. *C. parapsilosis *and *C. albicans *Cnh1 proteins are very efficient transporters and their capacities exceed that of *S. cerevisiae *Nha1p. On the other hand, the *C. dubliniensis *antiporter has a much lower transport activity and its ability to recognize and transport the smallest cation, toxic lithium, is very limited compared to the other two *Candida *antiporters (Figure 2, Table 3). The large difference between the transport activities of the evolutionary close *C. albicans *and *C. dubliniensis *Cnh1 antiporters is worth noting, as these two proteins shared the highest level of identity (Table 2). Their core membrane section (twelve tms and connecting loops) is almost 99% identical and their N-termini, composed of 11 aa residues, are 100% identical. These two antiporters differ slightly (compared to other antiporters) in the length and composition of their hydrophilic C-termini. Whether the observed transport capacities are based on the C-terminal difference remain to be established, though previous studies showed that the C-termini of yeast Na^+^/H^+ ^antiporters are not the most important factor in this activity [7] and that a single amino-acid exchange in one of the transmembrane domains can significantly influence both the substrate specificity and transport capacity of the antiporter [28,29].

Though the *C. parapsilosis *Cnh1 antiporter mediates a high and efficient sodium efflux from *S. cerevisiae *cells (similarly as the *Ca*Cnh1p, Figure 5 and Table 4), its physiological role in *C. parapsilosis *remains to be established. A recent study [20] showed that the Cnh1 antiporter is mainly important in potassium homeostasis in *C. albicans *cells and its role in Na^+ ^detoxification is rather marginal.

The differences observed in alkali-metal-cation tolerance between *C. dubliniensis *and *C. parapsilosis/C. albicans *species were also found upon testing the alkali-metal-cation tolerance of *S. cerevisiae *cells expressing the antiporters of these three species. Efflux measurements confirmed that the differing tolerances of *S. cerevisiae *cells were based on the differing transport activities of the *Candida *antiporters. Altogether, our results suggest that the activity of plasma-membrane Na^+^/H^+ ^antiporters is one of the factors determining the tolerance to high external concentrations of alkali metal cations in pathogenic *Candida *species.

## Methods

### Yeast strains, media and growth conditions

To determine the salt tolerance of various *Candida *species, *C. albicans *SC5314, *C. glabrata *ATCC2001, *C. dubliniensis *CD36 and *C. parapsilosis *CBS604 were used, together with *S. cerevisiae *S288c as a control. The *CdCNH1 *and *CpCNH1 *genes were isolated from *C. dubliniensis *CD36 and *C. parapsilosis *CBS604, and heterologously expressed in *S. cerevisiae *BW31a (*ena1-4*Δ *nha1*Δ, W303 derivative, [8]). Yeast cells were grown in YPD or YNB-NH_4_^+ ^media supplemented with 2% glucose at 30°C. Salts were added to the media prior to and auxotrophic supplements after autoclaving.

### DNA manipulations, plasmid construction and DNA sequencing

For DNA manipulations, standard protocols [30] were used. The *CdCNH1 *and *CpCNH1 *gene were amplified by PCR with platinum *Pfx *polymerase with proofreading activity (Invitrogen) using their isolated genomic DNA [31] as a template.

Plasmids for the heterologous expression of these antiporters in *S. cerevisiae *were constructed by homologous recombination in BW31a cells. The oligonucleotides used are listed in Table 5. Two types of plasmids were constructed. *CdCNH1 *and *CpCNH1 *coding sequences were cloned behind the *ScNHA1 *promoter either in multicopy YEp352 or in pGRU1, enabling C-terminal GFP tagging [7]. All constructs were analysed by sequencing in an ABI PRISM 3100 DNA sequencer using the BigDye Terminator v3.1 Cycle Sequencing Kit (Applied Biosystems).

**Table 5:** Oligonucleotides used for amplification of *C. dubliniensis *and *C. parapsilosis CNH1 *genes. Sections homologous to *CNH1 *genes are underlined.

Primer	Sequence (5'-3')
*CdCNH1*-F	TTTTTTGTACATTATAAAAAAAAATCCTGAACTTAGCTAGATCTTATGGCTTGGAGTCAGTTAGAA
*CdCNH1*-R	ACGACGTTGTAAAACGACGGCCAGTGCCAAGCTTGCATGCTACTCCTCTTCATCTTCTCT
*CdCNH1-GFP-R*	CATTTTAATAAAGCTCCGGAGCTTGCATGCCTGCAGGTCGACCTCCTCTTCATCTTCTCTATC
*CpCNH1*-F	TTTTTTGTACATTATAAAAAAAAATCCTGAACTTAGCTAGATCTTATGGTATGGAGTCAACTAGAA
*CpCNH1*-R	ACGACGTTGTAAAACGACGGCCAGTGCCAAGCTTGCATGCTATTCCTCGTCATTTTGATC
*CpCNH1-GFP-R*	CATTTTAATAAAGCTCCGGAGCTTGCATGCCTGCAGGTCGACTTCCTCGTCATTTTGATCACG

### Salt tolerance determination

The cell tolerance to various alkali metal cations was determined by drop test experiments. 3 μl of serial 10-fold dilutions of saturated cell cultures were spotted on solid media. For *Candida *species, YPD – 3% agar plates supplemented with increasing amounts of salts were used (800 – 2600 mM NaCl; 1800 – 2500 mM KCl; 20 – 1000 mM LiCl; 700 – 1800 mM RbCl). Determination of the maximum salt tolerance of *S. cerevisiae *BW31a cells expressing either of the antiporters was performed on solid YNB media supplemented with salts and adjusted to various pH levels as described previously [7]. The following salt concentrations were used: 1) non-adjusted pH, 500–1500 mM NaCl; 1800–2100 mM KCl; 25–40 mM LiCl; 1000–1600 mM RbCl; 2) pH 3.5, 500–1300 mM NaCl; 1600–2200 mM KCl; 20–50 mM LiCl; 3) pH 5.5, 200–1000 mM NaCl; 1900–2200 mM KCl; 15–40 mM LiCl; 4) pH 7.0, 50–200 mM NaCl; 800–1500 mM KCl; 5–10 mM LiCl.

### Cation efflux measurements

The transport activity of the antiporters was measured as the cation efflux from cells according to [7] with minor modifications. Cells were grown to OD_600 _≈ 0.2 in YNB, harvested and then a pH 5.5 incubation buffer (20 mM MES, supplemented with 10 mM KCl or 10 mM RbCl to prevent Na^+ ^or K^+ ^reuptake respectively) was used. For the determination of Na^+ ^efflux, the cells were preloaded for 60 min with 100 mM NaCl in YNB adjusted to pH 7.0 with NH_4_OH. The K^+ ^efflux was measured directly in the harvested cells. Samples were taken from cell incubation cultures at regular time intervals over a period of 60 min and the cellular cation content was determined by atomic absorption spectroscopy [7]. Each efflux experiment was repeated at least three times and representative results are shown.

### Microscopy analysis

Exponential phase cells (grown in YNB at 30°C, OD_600 _≈ 0.15) expressing the *CdCHN1 *or *CpCNH1 *gene tagged with the GFP sequence were viewed with an Olympus AX70 microscope using a U-MWB cube with a 450–480 nm excitation filter and 515 nm barrier filter. The micrographs were recorded with a DP70 digital camera using the program DP Controller. For whole-cell pictures, Nomarski optics was used.

### Immunoblotting

Exponetially growing (OD_600 _≅ 0.15) BW31a cells expressing GFP-tagged antiporters were harvested and concentrated by centrifugation to OD_600 _= 3.0. The proteins were extracted according to [32] with some modifications. After resuspension of cell pellet in 150 μl freshly prepared 1.85 M NaOH with 7.5% β-mercaptoethanol and incubation for 15 min on ice, 150 μl of cold 50% trichloroacetic acid were added. After incubation on ice for 20 min the collection of precipates by centrifugation at 20,000 × g for 20 min followed. The pellet was resuspended in 190 μl of 50 mM Tris-HCl buffer (pH 6.8), containing 8 M urea, 5% sodium dodecyl sulfate (SDS), 0.1 mM EDTA and 1.5% dithiothreitol (DTT) + 10 μl 1 M Tris base. After incubation for 30 min at 37°C the samples were centrifuged at 20,000 × g for 30 min. Supernatant (7.5 μl) were directly loaded on 8% glycine gel and separated by polyacrylamid gel electrophoresis (PAGE). Separated proteins were transferred via electroblotting on nitrocellulose membrane. To detect the GFP-tagged proteins on membranes, rabbit polyclonal anti-GFP antibody (Santa Cruz Biotech., diluted 1:200), secondary goat anti-rabbit IgG antibody with conjugated peroxidase (BioRad, diluted 1:10,000) and ECL detection kit (Pierce) were used.

## Authors' contributions

HS was involved in the design phase, YK provided the experimental data, and both authors drafted the manuscript, read and approved its final version.
